# Fabrication, characterization and evaluating properties of 3D printed PLA-Mn scaffolds

**DOI:** 10.1038/s41598-024-67478-9

**Published:** 2024-07-18

**Authors:** Sina Dehghan-Toranposhti, Rasoul Bakhshi, Reza Alizadeh, Mahboubeh Bohlouli

**Affiliations:** 1 Department of Materials Science and Engineering, Sharif University of Technology, Azadi Ave., Tehran, 11155-9466 Iran; 2 Department of Materials Science and Engineering, North Carolina State University, Raleigh, NC USA; 3 Department of Tissue Engineering and Applied Cell Sciences, School of Advanced Technologies in Medicine, Shahid Beheshti University of Medical Sciences, Tehran, Iran

**Keywords:** Biodegradation, Bone tissue scaffold, FDM, Manganese, PLA, 3D printing, Engineering, Materials science

## Abstract

Polylactic acid (PLA) based scaffolds have attained considerable attention in recent years for being used as biodegradable implants in bone tissue engineering (BTE), owing to their suitable biocompatibility and processability. Nevertheless, the mechanical properties, bioactivity and biodegradation rate of PLA need to be improved for practical application. In this investigation, PLA-*x*Mn composite filaments (x = 0, 1, 3, 5 and 7 wt%) were fabricated, characterized, and used for 3D printing of scaffolds by the fused deposition modeling process. The effect of Mn addition on the thermal, physical, mechanical, and structural properties, as well as the degradability and cell viability of 3D printed scaffolds were investigated in details. The obtained results indicate that the PLA-Mn composite filaments exhibit higher chain mobility and melt flow index values, with lower cold crystallization temperature and a higher degree of crystallinity. This higher flowability led to lower dimensional accuracy of 3D printed scaffolds, but resulted in higher interlayer adhesion. It was found that the mechanical properties of composite scaffolds were remarkably enhanced with the addition of Mn particles. The incorporation of Mn particles also caused higher surface roughness and hydrophilicity, a superior biodegradation rate of the scaffolds as well as better biocompatibility, indicating a promising candidate for (BTE) applications.

## Introduction

Fused deposition modeling (FDM) has emerged as a favorable technique that enables fabrication of the polymeric parts with higher printing speed and lower cost than other additive manufacturing (AM) methods. In this method, a filament made of thermoplastic polymers is melted and deposited layer by layer through the nozzle onto the printing bed for manufacturing an object from a computer-aided design (CAD) model. Nowadays, the FDM method is extensively used in different fields including bio-medical applications, while it is possible by the help of this method to easily make porous scaffolds and customized implants^1,2^.

Nowadays, 3D-printed temporary implants and scaffolds have been widely used for the treatment of bone defects and diseases, since they can be absorbed gradually by the human body during the healing process and thus, secondary surgeries can be avoided for their removal. This reduces not only the operation expenditures, but also the pain and health issues during the healing period^3^. Polylactic acid (PLA) is among the biodegradable polymers which is approved by the food and drug administration (FDA) of the united states for the orthopedic applications, i.e., scaffolds, screws and drug delivery^4^. Over the past few years, PLA-based scaffolds have grabbed increasing attention for tissue engineering applications owing to their biocompatibility, bioactivity and suitable processability^5^. Nevertheless, pure PLA scaffolds suffer from slow degradation rate, poor mechanical properties and inherent hydrophobicity, which all prevent their extensive use in some biomedical applications^6,7^. Moreover, the biocompatibility of PLA should be improved, as the acidic components produced during the degradation process would be adverse to the viability of cells^8^.

In order to address the mentioned challenges for the PLA-based scaffolds, numerous studies have been conducted on the incorporation of different fillers in the PLA matrix. In this regard, different PLA-metal composites with prominent properties have been developed and studied in recent years^9^. For example, it has been reported that addition of magnesium (Mg) particles into the PLA accelerated the degradation rate of scaffolds in body fluids and improved cellular activity as well as the mechanical properties of scaffolds^10^. However, rise in volume fraction of Mg in the PLA matrix can bring about rapid degradation rate, that increases the amount of evolved hydrogen and also the pH level, which are all detrimental to cell viability^11–13^. Compared with PLA/Mg composite scaffolds, the incorporation of iron (Fe) particles in PLA exhibited superior strength and lower changes in the pH of the used saline solution without local toxicity. In contrast, the slow corrosion rate of pure Fe makes it not an appropriate choice where biodegradation in reasonable times is of great importance and priority^14,15^.

Manganese (Mn), as a biodegradable metal, can be an interesting option to overcome the mentioned challenges. Mn is an essential mineral for the body and is responsible for different metabolic functions, including those involved in nervous system function, reproductive hormone functions and in antioxidant enzymes that protect cells from damage due to free radicals. This element participates as a cofactor of a wide variety of enzymes and plays an important role in the regulation of cellular bone, tissue growth and blood clotting. Mn deficiency can impair the bone formation and disrupt the function of reproductive and nervous systems^16^. Furthermore, Mn has demonstrated good biocompatibility and offered a suitable biodegradation rate intermediated to that of Mg and Fe. In fact, Mn has a lower and a higher standard electrode potential (− 1.18 V) than Fe (− 0.44 V) and Mg (− 2.37 V), respectively^17^.

Up to now, the development of PLA-Mn composite filament and scaffolds via FDM method has not been reported yet and thus this is the main goal of this study. In his regard, PLA filaments with different loadings of Mn are prepared and analyzed. This investigation provides an in-depth understanding of influence of Mn particles on the mechanical properties, wettability, water absorption, biodegradation, and thermal behavior of 3D-printed scaffolds.

## Materials and methods

### Filament fabrication

Detailed explanation about the method used for fabrication of composite filaments in this investigation are given in our previous works^18,19^, and would be referred only briefly here. In the first step, pure Mn powder (with spherical shape and particle size lower than 45 μm, Merck, Germany) was added to dichloromethane (DCM) (Neutron pharma chemical company with a 99.8% purity) and stirred at room temperature for 5 min. The prepared blend was poured on the PLA granules (4043D, Ingeo™ Biopolymer) and simultaneously mixed mechanically for 10 min, ensuring that a uniform mixture was obtained. Afterward, the produced mixture (Mn content = 0, 1, 3, 5 and 7 wt%) was heated and dried in an oven at 80 ℃ for 12 h for complete elimination of moisture and DCM. Finally, the dried mixture was fed into an extruder at 195 ℃ with the average screw rotation speed of 30 rpm for the production of PLA and PLA-Mn filaments, with the diameter of 1.75 ± 0.10 mm.

### 3D printing

**Figure 1:**
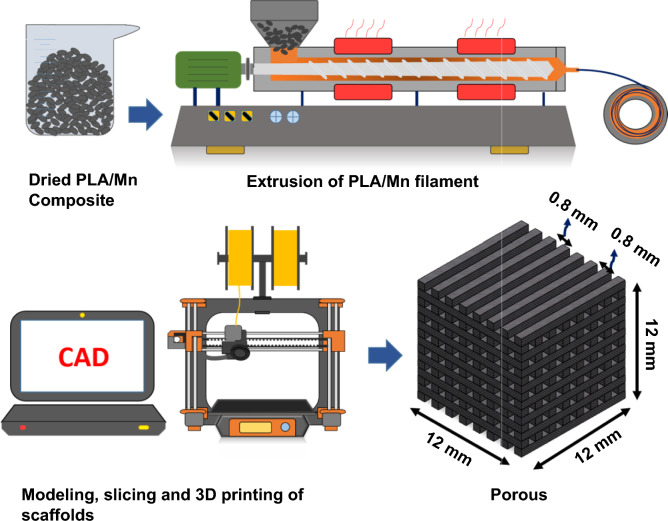
Schematic representation of the process used to print composite PLA-Mn composite scaffolds.

A desktop FDM 3D printer (Dayan K12S, Borna3D, Iran) with a single extruder nozzle was employed for scaffold fabrication. The structure of porous scaffolds was first designed by SolidWorks. Then, the CAD model was converted to a STL file, which was further sliced by the Mankati software and at the end, the G-code was produced for printing. In this investigation, cubic scaffolds with length of 12 mm, porosity of 47%, pore size of 0.8 mm and strut size of 0.8 mm were fabricated, using printing parameters listed in Table 1. All fabrications steps used in this investigation to 3D print PLA-Mn scaffolds are schematically shown in Fig. 1.

**Table 1:** FDM process parameters for printing PLA-Mn composite scaffolds.

Parameter	Quantity
Printing speed	30 mm/s
Nozzle temperature	210 ℃
Bed temperature	60 ℃
Nozzle diameter	0.4 mm
Layer height	0.2 mm
Infill density	100%
Infill pattern	0°/90°

### Filament characterization

Quality of the fabricated composite filaments was evaluated in terms of distribution of Mn particles and existence of porosity, using field emission scanning electron microscopy (FESEM, TESCAN MIRA 3 LMU). A thin layer of gold was deposited on the samples, utilizing a sputter coater, to make them electrically conductive for FESEM observations. The thermal properties of filaments were evaluated by differential scanning calorimetry (DSC, Q100, TA Instruments, USA), where glass transition temperature (*T*_g_), cold crystallization temperature (*T*_cc_), melting point (*T*_m_), cold crystallization enthalpy (*H*_cc_), and fusion enthalpy (*H*_m_) were determined. Samples with a weight of 5–10 mg were sealed in the aluminum pan. For DSC tests, about 5–10 mg of each material was placed in an alumina pan, and then the test was run on heat-cool-heat cycles from 40 to 180 °C with a heating and cooling rate of 10 °C/min under nitrogen flux. The overall degree of crystallinity (*X*_c_) was also determined as

1 $${\rm X}_{C}\left(\%\right)=\frac{\Delta {H}_{m}}{\Delta {H}_{0}\times {W}_{PLA}} \times 100 \%$$

where $\Delta {\text{H}}_{0}$ = 93.7 J/g is the melting enthalpy of 100% crystalline PLA, and W_PLA_ refers to the weight fraction of PLA in the composite filament^14,20^.

For evaluating the effect of Mn particles on the flowability and printability of the composite filaments, the melt flow index (MFI) test was conducted according to the ASTM D1238 standard. According to this standard, a load of 2.16 kg was applied on a plunger for 10 min at barrel temperature of 190 °C.

### Mechanical properties

The compression test was carried out at a constant crosshead speed of 1 mm/min using a universal Santam testing machine on 3D-printed composite scaffolds in accordance with the ASTM D695 standard. Tests were repeated for four times on each material, and then the average values of the ultimate compressive strength (UCS), elastic modulus and stiffness were obtained from the stress–strain curves. To further investigate the main mechanical failure mechanisms of the scaffolds, surface morphology of the deformed scaffolds after compression test was studied by FESEM.

### Water absorption

The water absorption capacity was measured based on the ASTM D570 standard in order to evaluate the hydrophilicity and the capillary of the fabricated scaffolds^21^. For this purpose, initially, the scaffolds were weighed with an accuracy of ± 0.0001 g and then immersed in distilled water for time intervals of 24, 48, and 72 h, at 37 ℃. After each time span, samples were removed from water and weighted. The water absorption capacity of scaffolds was calculated by the following equation:

2 $$Water absoption capacity \left(\%\right)=\frac{{W}_{s}- {W}_{d}}{{W}_{d}}\times 100$$

where *W*_s_ and *W*_d_ are the weight of samples before and after immersion, respectively.

### In-vitro biodegradation tests to measure the ion release and pH changes

Biodegradation test was performed in the phosphate-buffered saline (PBS) solution at 37 ± 0.5 ℃. The chemical composition of the used PBS solution was 8.0 g/L NaCl, 1.15 g/L Na_2_HPO_4_, 0.2 g/L KCl, and 0.2 g/L KH_2_PO_4_ in distilled water. For this purpose, four scaffolds of pure PLA and PLA-7Mn composite were weighed and immersed in the PBS solution weekly for a period of 10 weeks. After immersion, samples were dried in an oven at 80 ℃ for 3 h, following the procedure suggested in^10^. The weight of samples was calculated after each time span and the average weight of each sample group was reported accordingly. The degradation rate was determined using the following equation:

3 $$Weight loss \left(\%\right)=\frac{{W}_{I}- {W}_{0}}{{W}_{0}}\times 100$$

where *W*_I_ and *W*_0_ are the initial and degraded weight of the scaffolds, respectively. Furthermore, to study better the degradation behavior of different samples, the surface morphology of specimens was observed by FESEM after 4-week immersion.

Additionally, concentration of the released Mn ion from scaffolds immersed in the PBS solution was measured via flame atomic absorption spectroscopy (AAS), following the methodology outlined in^22^. In this regard, 5 ml of solution was extracted and prepared at intervals of 2, 4, 6, 8 and 10 days. After each collection, the same amount of PBS was added to the main degradation medium to maintain the previous condition. Moreover, to follow the pH changes during immersion and degradation, the pH value of the solution was measured and recorded with the use of a pH meter (Shimaz, Iran) for up to 4 weeks.

### Contact angle measurement

Contact angle measurement (DATA physics OCA15 plus, Germany) was performed at three different locations of the PLA and PLA-7Mn samples, and average values were reported. In this technique, a 2 µL droplet of distilled water was placed on the sample using a microliter syringe, and the contact angle with tangent method was measured.

### Cell isolation and culture

Adipose tissue-derived mesenchymal stem cells were obtained from stem cells bank of Shahid Beheshti University of Medical Sciences that were harvested from abdomen region of three healthy donor (100% male, 25–35 years) during liposuction surgery. The cells were isolated using enzymatic (collagenase type I) protocol and cultured in standard medium containing high-glucose Dulbecco's Modified Eagle Medium (DMEM;Gibco), 15% fetal bovine serum (FBS; Gibco), and 1% penicillin and streptomycin antibiotic (pen/strep; Gibco) in T-25 flasks under 5% CO_2_ at 37 ℃. Afterwards, MSCs were seeded on the sterilized scaffolds at a density of 1 × 10^6^ cells/cm^3^ at three passage number.

### Cytotoxicity evaluations

Cytotoxicity of the scaffold were assessed using 2,5-diphenyl-2H-tetrazolium bromide (MTT) at 1, 3, 5, and 7 days. For this procedure, fresh medium containing 1% MTT solution (Sigma) was added and scaffolds were incubated at 37 ℃ for 3 h. The formazan crystals were dissolved in 500 µl DMSO after removing medium. The absorbance was measured at wavelength of 570 nm with an ultra-microplate reader. Furthermore, Scaffolds containing MSCs were treated with 2 µM Acridine Orange and 4 µM Propidine Iodide (PI) in 200 µl of PBS solution, followed by a 1-h incubation at 37 ℃. Staining was observed under the inverted fluorescence microscope, then live and dead cells were represented in green and red fluorescence, respectively.

### Ethical approval and informed consent

No human or animals used in this study.

## Results and discussion

### Characterization of the PLA-Mn composite filaments

#### Thermal characterization

**Figure 2:**
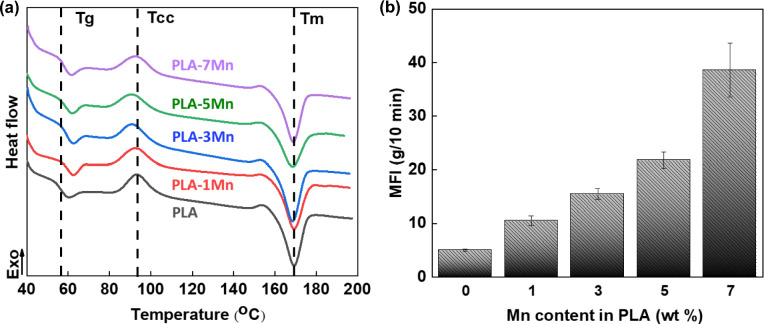
(**a**) DSC curves, and (**b**) MFI results for the PLA-Mn composites with different Mn contents.

The thermal properties of PLA and PLA-Mn composites were studied by DSC, and their profiles at the second heating cycle are depicted in Fig. 2a. Three distinct phase transitions corresponding to *T*_g_, *T*_cc_, and *T*_m_ are shown and compared in this figure. The data of the second heating cycle are reported in order to remove the thermal history (Table 2). It can be observed that while the glass transition temperature of pure PLA was 61.4 ℃, the *T*_g_ of the composite filaments remained almost unchanged with an increase in Mn content. Chain mobility, steric impacts, intermolecular interactions, and molecular weight (MW) are some important factors which directly can affect *T*_g_^23^. It can be supposed that decrement of MW and higher mobility of polymeric chains are induced by chain scission and depolymerization during the extrusion process, which resulted in lower values of *T*_g_^24^. In addition, the entanglement effect is believed to be reduced in the vicinity of the non-interacting interface of the polymer-based composite, which can increase the chain mobility, resulting in the *T*_g_ decrement of the composite^25,26^. On the other hand, the presence of metallic fillers may restrict the movement of PLA chains, which can cause a decreased lubrication effect of PLA chains and thus a higher temperature of glassy transition^27^. Consequently, stability of the *T*_g_ could be attributed to the balance between the confinement effect of filler particles on the mobility of polymer chains and the plasticizer effect of chain scission.

**Table 2:** The obtained data from the DSC analysis.

Samples	T_cc_ (℃)	ΔH_m_ (J g^-1^)	X_c_ (%)
PLA- Pure	123.4	35.77	38.17
PLA-1Mn	122.5	36.13	38.95
PLA-3Mn	120.5	37.97	41.78
PLA-5Mn	117.6	38.86	43.65
PLA-7Mn	117.2	38.98	44.73

According to the obtained results, the cold crystallization temperature declined noticeably from 123.4 ℃ for neat PLA to 117.2 ℃ for the PLA-7Mn filament, which can be explained by two mechanisms. First, Mn particles can act as a nucleating agent that may reduce the barrier energy for nucleation and facilitate crystallization at lower temperature upon heating^25^. Second, thermal and shear degradation of polymer during extrusion produces little and more flexible chains, and this phenomenon can be catalyzed by the incorporation of Mn particles in the PLA matrix^28,29^. These smaller molecules can move and rearrange themselves easier in crystalline lamellas during heating which leads to the decline of *T*_cc_ in the PLA-Mn composites. Moreover, the degree of crystallinity of samples is shown in Table 2. The slight increase in crystallinity confirms previous claims about the nucleation effect of Mn in the PLA matrix and the higher mobility of chains. A similar observation was reported in other researches^27,30^. The degree of crystallinity can affect mechanical properties of 3D-printed scaffolds, which will be discussed with details in the following parts.

The melting temperature peaks are identified to be approximately at 167 ℃, for all studied materials. The size and perfection of the crystalline lamellae are two important parameters which can affect *T*_m_^31^. As a result, it can be proposed that the crystalline structure of PLA filament has remained unchanged after the addition of Mn particles. Additionally, the melting peak of PLA-7Mn composite shows slight broadening. This melting behavior has been associated with a melt-recrystallization-melt mechanism, where the small or defective crystals (due to cold crystallization) melt initially at lower temperatures, reorganize in more stable and perfect crystals during the heating scan, and then melt at higher temperatures^32,33^.

#### Melt flow index characterization

Melt flow index of the studied materials are summarized in Fig. 2b. MFI is a characteristic feature which can be used for the evaluation of the printing quality of molten composites. The MFI can have an effect on the process precision and indeed, on adhesion between the layers of the printed specimen, which itself can greatly influence mechanical properties of printed parts. In order to have a suitable printing process, molten flowability should be high enough to prevent the nozzle blockage and low enough to sustain its weight and to provide shape stability of filament during the deposition stage^34^.

As it can be noted in Fig. 2b, the incorporation of Mn particles in PLA increased significantly the MFI of the composite from 5 g/10 min in pure PLA to 39 g/10 min in PLA-7Mn composite. This observation is in complete agreement with the assumption of molecular weight loss and confirms the catalytic effect of Mn particles on chain scission and depolymerization during the extrusion process, as discussed before. Thus, the higher number of short chains of PLA in composites, which have higher mobility, seem to enhance the MFI. According to previous studies^34,35^, the printability window of the filament should be between 10 and 30 g/10 min to have a successful printing process by the FDM method, however, printing process can be possible also out of this range, but with lower quality. Accordingly, it was reported that the MFI of PLA filaments with 10.5, 14, and 17.5 wt% of Zn were out of this range and these composite filaments failed to be printed successfully. In the current study, although the MFI of PLA-7Mn composite was slightly out of the mentioned range, the designed samples could be successfully printed with acceptable accuracy.

#### Fourier transform infrared spectroscopy (FTIR)

**Figure 3:**
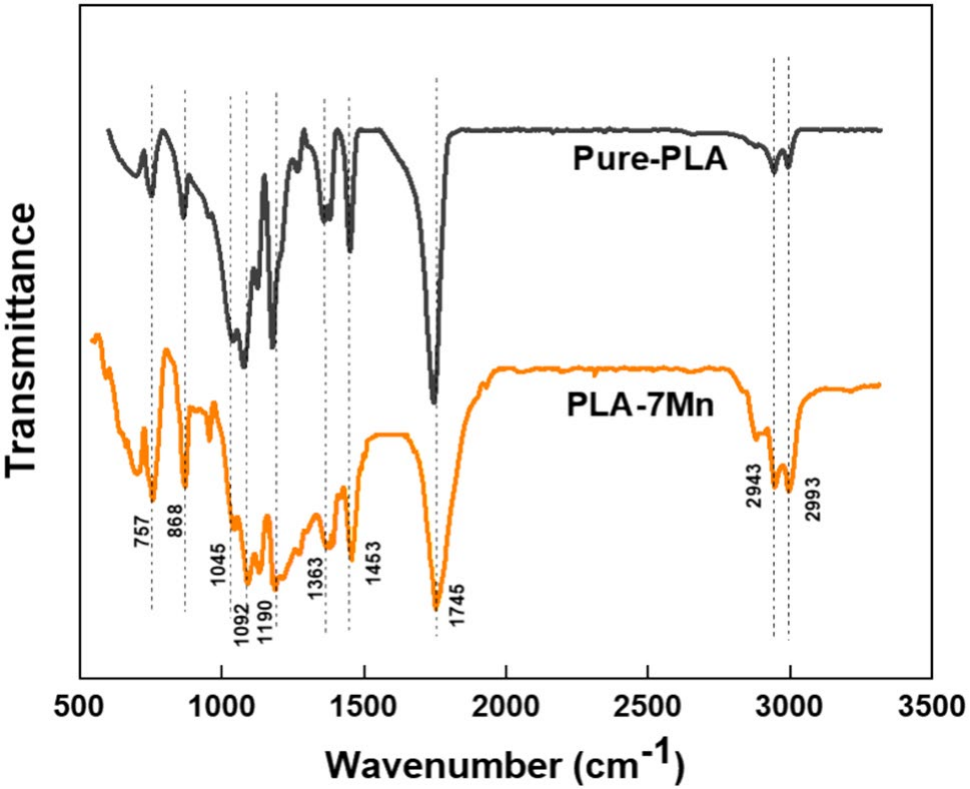
FTIR spectra of pure PLA and PLA-7Mn composite.

To determine the existence, type and degree of interaction between PLA and manganese, FTIR spectroscopy was performed on the PLA-7Mn composite and compared with the pure PLA. As shown in Fig. 3, the major detected peaks are described as the lowest to highest wavenumber. These peaks are observed at 1092 and 1190 cm^−1^ for the –O–C=O bonding and stretching –CH–O bonding, respectively. The peaks at about 1363, 1453 and 1745 cm^−1^ belong to C–H stretching, CH_3_ and C=O bonding, respectively^19^. The peaks at 2943 and 2993 cm^−1^ are associated with asymmetric and symmetric stretching of C–H bonding. It can be observed that the FTIR spectrum of PLA-7Mn is almost similar to that for pure PLA, indicating that Mn does not form any chemical bonds with PLA and particles are physically interspersed among the PLA chains. A slight peak broadening could be seen in the carbonyl functional (C=O) of PLA-7Mn at a wavenumber of 1745 cm^−1^ which is attributed to degradation of polymers^36^.

#### FESEM characterization

**Figure 4:**
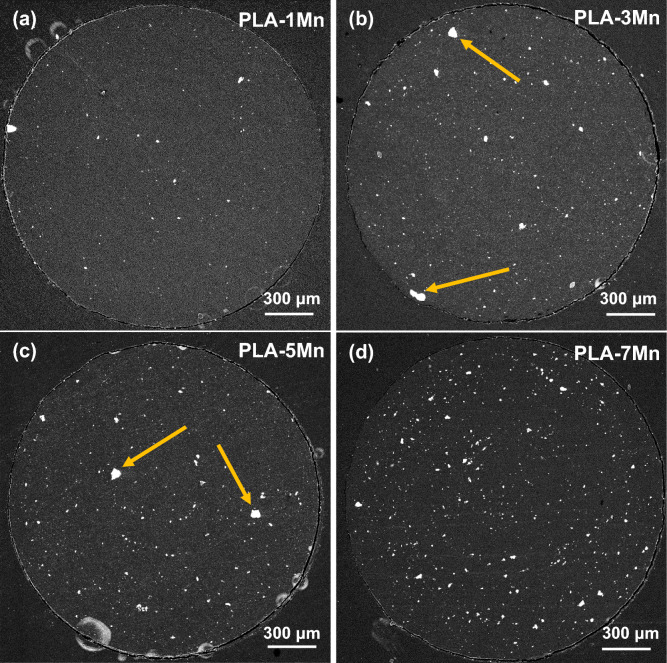
FESEM cross section of: (**a**) PLA-1Mn, (**b**) PLA-3Mn, (**c**) PLA-5Mn, (**d**) PLA-7Mn composite filaments.

Figures 4 and S1 (Supporting Information) demonstrate the FESEM analysis and EDS elemental mapping conducted on the circular cross-section of the extruded composite filaments with various loading of Mn particles, respectively. It can be noted that the distribution of these micro-particles becomes more homogeneous with an increment of the Mn content. Some agglomeration can be observed in the PLA-3Mn and PLA-5Mn filaments indicated by arrows (Fig. 4b,c), while the severity of agglomeration decreases in the PLA-7Mn filament (Fig. 4d). According to the previous works^37,38^, this can be attributed to the increase flowability of PLA with the incorporation of Mn, which provides better wetting and interaction between filler and polymer chains (owing to higher chain mobility). Consequently, homogeneous distribution of filler during extrusion is obtained in the highest content of Mn.

**Figure 5:**
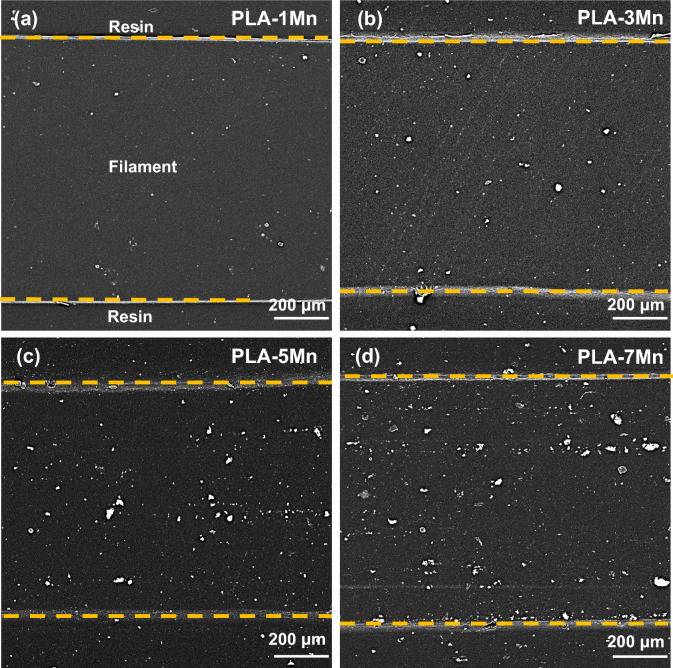
FESEM longitudinal cross section of: (**a**) PLA-1Mn, (**b**) PLA-3Mn, (**c**) PLA-5Mn, (**d**) PLA-7Mn composite filaments.

In addition to the cross section studies perpendicular to the filament axis, features of longitudinal cross sections are also depicted in Fig. 5. As it is evident in this figure, the extent of preferential alignment of Mn particles increases with increasing the Mn content. It is stated that the filler particles experience high shear strains in the extrusion die during the fabrication process^39^. Additionally, a higher concentration of particles can be observed near the center of filaments rather than close to the wall. This event indicates higher tendency of the Mn particles to migrate toward the centerline, which was reported in other studies as well^40,41^, and it is ascribed to the radial forces and small blockage ratios (size of the particle divided by the size of the extruder channel) that force the filler particles to migrate toward the center during extrusion. Moreover, no pores, voids and moister bubbles can be found in both circular and longitudinal cross sections (Figs. 4, 5). The perfect drying of composite pellets before the extrusion process and great integration and adhesion of Mn particles in the PLA matrix can prevent pore and bubble formation in the filament.

**Figure 6:**
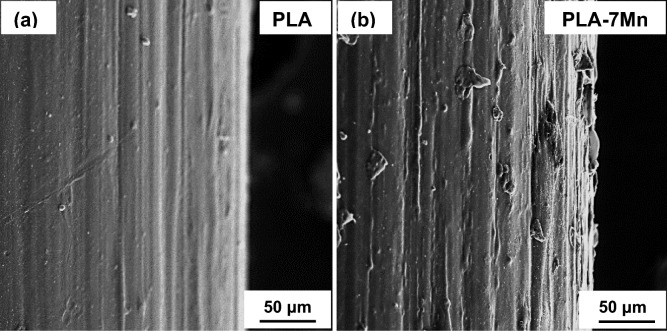
FESEM micrographs showing the surface roughness of fabricated filaments: (**a**) pure PLA, and (**b**) PLA-7Mn.

Surface roughness of the neat PLA and PLA-7Mn filaments can be compared based on the FESEM micrographs provided in Fig. 6, where it can be observed that the outer surface of pure PLA filament is relatively smooth (Fig. 6a), whereas the PLA-7Mn exhibits a rough surface which can be ascribed to the presence of Mn micro-particles (Fig. 6b). The increase of filament surface roughness with the addition of metallic filler is a common phenomenon and has been reported in other studies^10,42^. The surface roughness of filaments can influence directly the surface roughness of 3D-printed scaffolds, which will be discussed in the next section.

### Characterization of the 3D printed PLA-Mn composite scaffolds

#### Structural characterization

**Figure 7:**
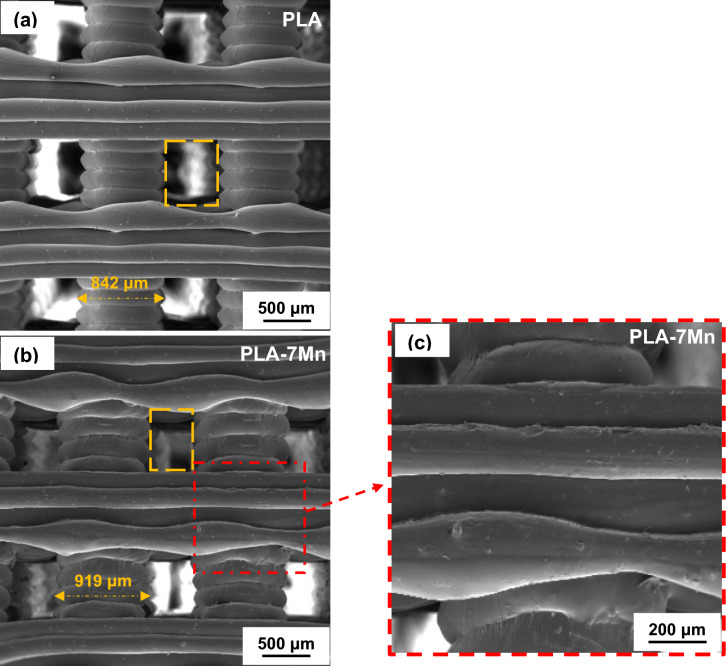
FESEM micrographs of the fabricated scaffolds: (**a**) PLA, and (**b**, **c**) PLA-7Mn.

In addition to the quality of the made filaments, it is also of great importance to evaluate the quality of 3D printed parts from the viewpoints of homogeneity, distribution of Mn particles, and possible presence of cracks, voids or other printing defects. In this regard, FESEM micrographs of the 3D printed PLA and PLA-7Mn scaffolds are shown in Fig. 7. By comparing Fig. 7a,b, it is revealed that the PLA-7Mn scaffold exhibits a rougher surface in comparison with the neat PLA sample, similar to the trend observed for filaments. It is noticeable that several cracks and chips can be seen on the outer surface of the composite struts in the magnified image shown in Fig. 7c. This phenomenon can be associated with the friction in the vicinity of the nozzle and filament throughout the extrusion, as similarly has been in the literature of^15^. Also, the presence of Mn particles embedded in the PLA matrix is visible on the struts as stiff locations which caused higher surface fluctuations than neat PLA struts. The reason for this observation is that the fillers tend to aggregate at the nozzle of the printer^43^. Generally, it is found that^44^ a rougher surface promotes cell attachment and proliferation. Hence, it can be anticipated that PLA-7Mn scaffolds should show better compatibility for tissue engineering applications.

Furthermore, it is clear that the dimensional accuracy and printing quality of the PLA-7Mn scaffold is lower than the neat PLA sample. According to Fig. 7a, the PLA scaffold has a deposited layer width of 837 μm, which is slightly over the CAD designed value of 800 μm, while the layers in the PLA-7Mn became significantly wider with an average value of 912 μm. Also, the square shape of the pores appeared in the PLA scaffold turned into a rectangular state in the PLA-7Mn scaffold. This observation is a result of the excessive flowability of PLA-s7Mn composite. Although the higher MFI facilitates processability and extrusion of the filament from the nozzle, the shape stability of the deposited layers of polymer would be reduced due to the higher mobility of polymer chains. Consequently, the struts become wider and more deformed under the stress generated by the weight of the subsequent upper layers deposited during printing^45^. On the other hand, better interlayer adhesion and no delaminated parts can be seen in the PLA-7Mn scaffold (Fig. 7b,c), which would play a positive role in controlling the mechanical properties of the 3D printed scaffolds^45^.

#### Mechanical behavior

**Figure 8:**
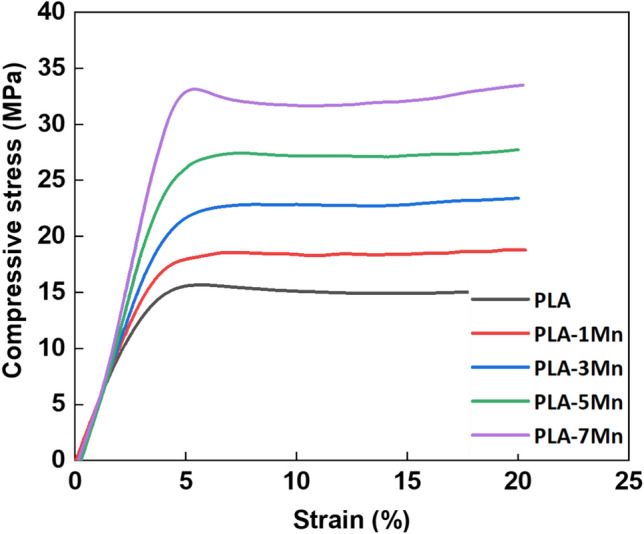
Compressive stress–strain curves of 3D printed PLA-Mn scaffolds.

**Figure 9:**
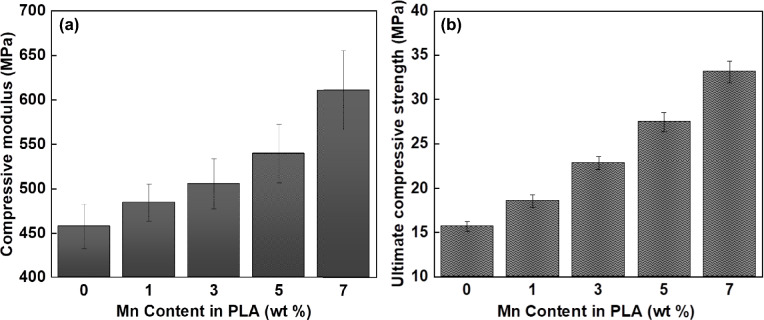
Summary of the obtained results from compression testing of PLA-Mn scaffolds: (**a**) compressive modulus, and (**b**) ultimate compressive strength.

Representative compressive stress–strain curves of the FDM-fabricated scaffolds are demonstrated in Fig. 8, and the corresponding compressive modulus and ultimate compressive strength (UCT) values are summarized in Fig. 9. According to the obtained results, it is clear that Mn addition has remarkably boosted the mechanical properties of PLA scaffolds. Interestingly, the addition of 7 wt% of Mn approximately doubled the UCS value, from 15.67 MPa for pure PLA to 33.13 MPa in the case of the PLA-7Mn composite. Likewise, compressive modulus of the composite scaffold witnessed a 33% improvement with the incorporation of 7 wt% of Mn in comparison with pure PLA, which increased from 457 to 611 MPa. The observed enhancement in mechanical properties of the scaffolds can be explained from the viewpoints of the direct reinforcing effect of Mn particles for PLA, and also, from the indirect effect of Mn particles on improved quality of printed parts. It is generally assumed that good adhesion between the PLA matrix and metal filler as a strong reinforcing phase can assist load transfer between the filler and matrix, which causes higher compressive properties. This has been observed in previous studies as well, that the addition of metallic fillers such as Mg, Ti, Ti64, Fe, and 316L stainless steel also improved the compression modulus and ultimate strength^10,15,19,46^.

**Figure 10:**
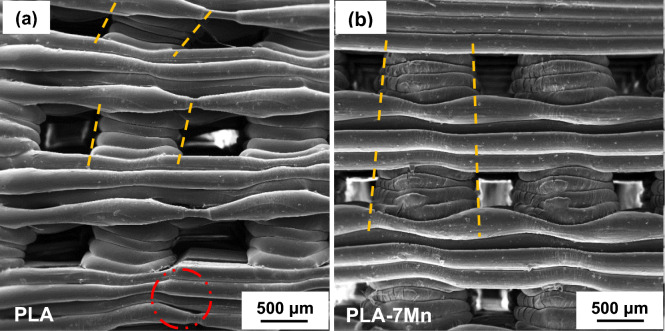
FESEM micrographs obtained from the lateral view of the deformed 3D printed scaffolds after compression test: (**a**) PLA, and (**b**) PLA-7Mn.

To further understand the compression behavior of the studied materials, FESEM micrographs of the deformed PLA and PLA-7Mn scaffolds after compressing for 20% are presented in Fig. 10. It can be observed that layer detachment has occurred in some parts of the PLA scaffold, shown by a red dashed circle (Fig. 10a), whereas the PLA-7Mn revealed a uniform deformation and no detachment could be detected (Fig. 10b). This behavior of pure PLA scaffold is related to the poor adhesion between struts. As discussed in the previous section, the addition of Mn particles to PLA has caused better interlayer bonding which helps to enhance the load-withstanding capacity of scaffolds^45^. Additionally, the strut bending, slipping and shape distortion in the intersection between the deposited layers are visible in the PLA scaffold, while the PLA-7Mn scaffold experienced a uniform deformation parallel to the stress direction, without any indications of these problems. It is proposed by the literature that the thermal shrinkage of PLA during the cooling process of the deposited material can induce some shape distortion in the place of layers intersection, where residual shear stresses can be localized and lead to structural distortions^47^. Conversely, metallic powders can prevent the thermal shrinkage of PLA after the deposition. As a result, the PLA-Mn composite scaffolds can present superior mechanical strength under compression.

#### Wettability and water absorption capacity

**Figure 11:**
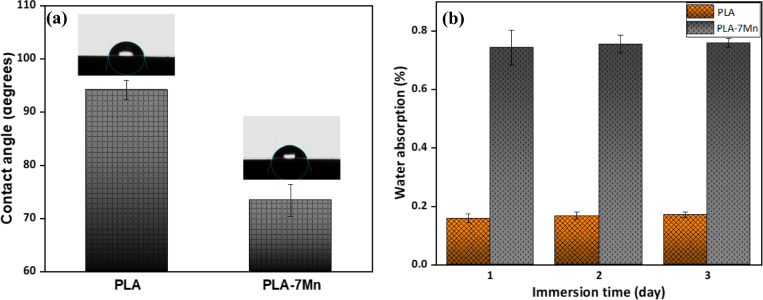
The water contact angle (**a**), and the water absorption capacity (**b**) of the PLA and PLA-7Mn scaffolds.

The surface wettability is a critical feature of biomaterials, defined as the ability of a liquid to spread over the surface, which plays an important role in biocompatibility and cellular behavior of manufactured scaffolds. A hydrophilic environment can increase the osteointegration and bone regeneration process due to the improved cell attachment and proliferation^48,49^. In this regard, contact angle and water absorption tests were performed on the PLA and PLA-7Mn scaffolds, and the related results are shown in Fig. 11.

The obtained results indicate that the addition of Mn particles has reduced the contact angle of PLA from 94.1° (hydrophobic surface) to 73.4° (hydrophilic surface) for the PLA-7Mn composite, according to Fig. 11a. Thus, the incorporation of Mn particles improves wettability of PLA significantly. This observation could be explained by the higher polarity of the scaffolds surface. As discussed before in the DSC analysis, depolymerization of PLA can produce the higher number of little chains with more polar functional groups, such as carboxyl and hydroxyl^28^. The presence of these hydrophilic groups in composite could favor the interactions between the water molecules and polymer and increase the surface hydrophilicity of 3D-printed scaffolds. Furthermore, the augmented surface roughness and higher number of pores on the PLA-7Mn scaffold surface, which was discussed before, can lead to higher exposure of water to the scaffold surface.

In close relation with the contact angle results, the PLA-7Mn scaffolds also revealed a higher water absorption capacity than the neat PLA scaffolds (Fig. 11b). The water absorption capacity of the PLA scaffold saturated after 2 days, while this saturation occurred within 1 day for the PLA-7Mn scaffold. The higher water absorption capacity of the composite scaffolds may be due to the increased wettability, interlayer voids and the presence of interconnected pores network that allow scaffolds to hold more amount of water^50^. In general, it can be concluded that the addition of Mn to the PLA matrix increases the wettability and water absorption capacity of PLA, which can result in the improvement of cell adhesion and proliferation on the surface of the fabricated scaffolds.

#### In-vitro biodegradation

**Figure 12:**
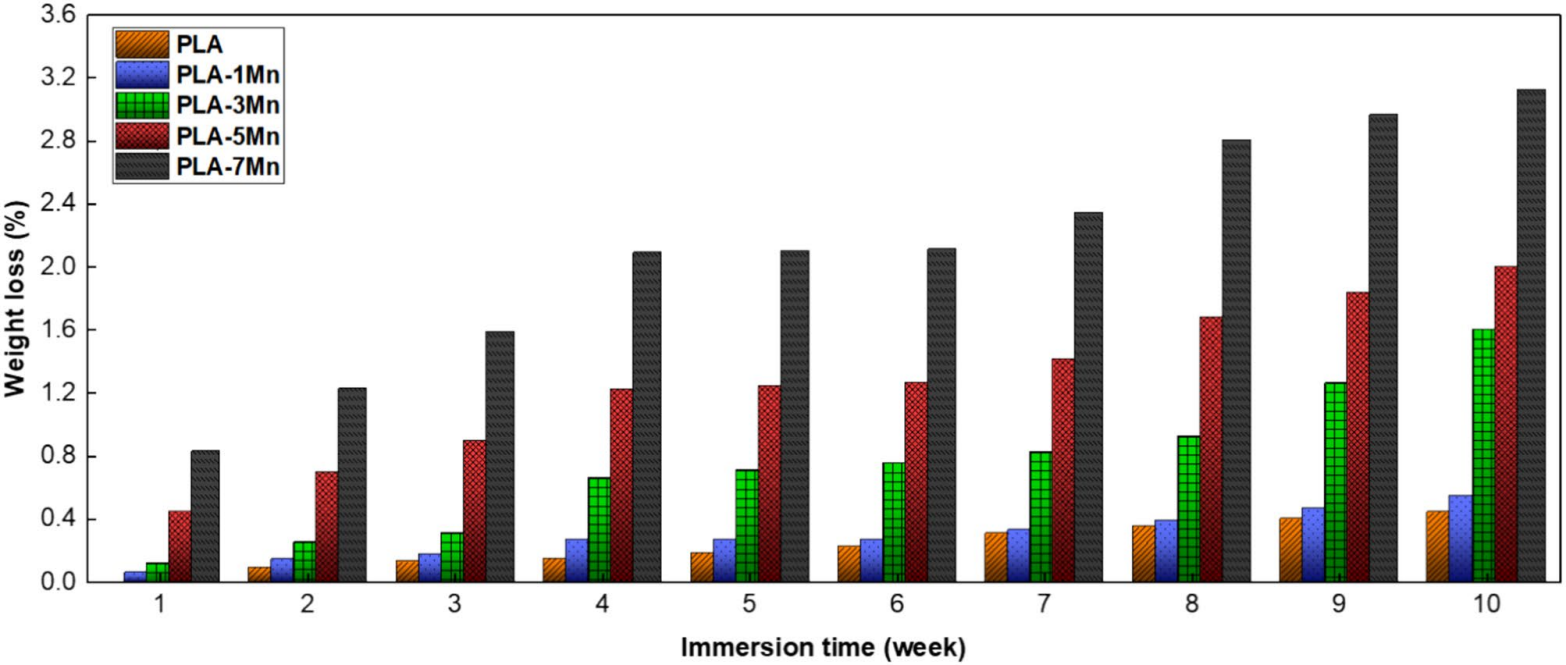
Comparison of the weight loss results for the PLA, PLA-1Mn, PLA-3Mn, PLA-5Mn, and PLA-7Mn scaffolds, during immersion in the PBS solution at 37 °C for 10 weeks.

A critical feature of all biodegradable implants is appropriate degradation rate, according to the regeneration rate of the proposed tissue. The slow pace of degradation and poor bioactivity, are two major limitations on the way of using PLA as a temporary template for hard tissue regeneration and integration^51^. In this regard, active metallic fillers, such as Mg, have been reported to be able to accelerate the degradation rate of PLA^10,52^, an effect which is further studied in the present work to understand the effect of various content of Mn on PLA degradation. Accordingly, the biodegradation rate of PLA and PLA-Mn composite scaffolds was measured and compared, and the corresponding results are displayed in Fig. 12. It is noticeable that PLA-Mn composite samples experienced a higher loss of weight and faster degradation rate than pure PLA scaffold during 10 weeks. Moreover, the difference between the levels of weight loss of scaffolds escalated with the increase in the load amount as the PLA-7Mn showed the highest weight loss with 3.13% at the end of the test period, while the PLA scaffolds reached to a low level of 0.44%.

Generally, the PLA degradation process begins with the random hydrolysis of ester bonds and then, the short chains and small units appear and the degradation products are formed which leak into the PBS solution, thus resulting in weight loss of the sample. However, the pace of this process is too slow, while the main factors influencing this rate are the molecular weight of polymer, morphology and porosity of samples, and temperature and pH of solution^53^. Conversely, as pointed out before, Mn particles increase the surface roughness, number of pores, and wettability of scaffolds, which all facilitate medium diffusion into the sample and cause higher exposure with the PBS solution. Accordingly, this phenomenon leads to the faster rate of hydrolytic degradation with the passage of time. Furthermore, Mn particles degrade and produce soluble compounds (hydroxide and phosphate-based compounds), which could leak into the solution and help further reduction of sample weight. In another study^54^, it was mentioned that a high concentration of the produced hydroxide ions can intensify the hydrolytic degradation of PLA due to local alkalization in the polymer/metallic filler interface. In other words, the PLA ester bonds in a higher alkaline environment are more susceptible to being attacked, an effect which results in higher degradation rates^53^.

**Figure 13:**
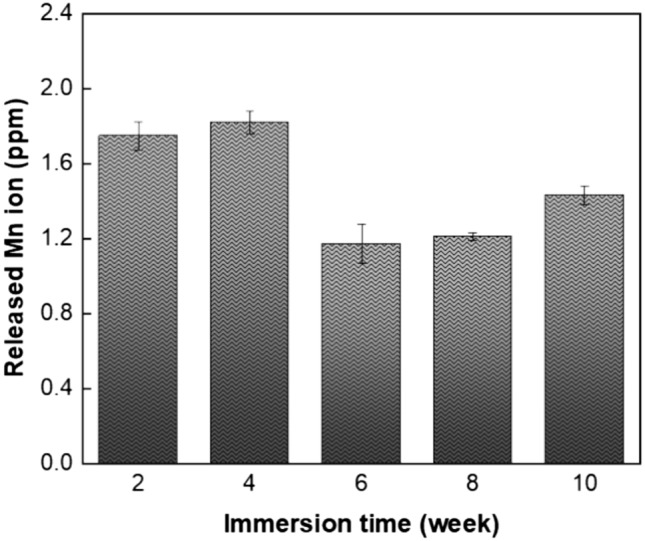
Evolution of Mn ion released from the PLA-7Mn scaffold into the PBS solution during long-term immersion up to 10 weeks.

Furthermore, in order to investigate the effect of Mn on the biomineralization of the scaffold surface, release of Mn ion from the PLA-7Mn scaffold into the medium was monitored every 2 weeks throughout the immersion period. According to Fig. 13, the concentration of Mn ion in PBS initially increases rapidly after 4 weeks, in accordance with the degradation results. Afterwards, concentration witnessed the lowest point after 6 weeks and then gradually increases until the end of the immersion duration. This sudden decline in concentration can be attributed to the consumption of Mn ion for the formation and deposition of hydroxide and phosphate base apatite on the scaffold surface, in accordance with previous works^12,55^.

**Figure 14:**
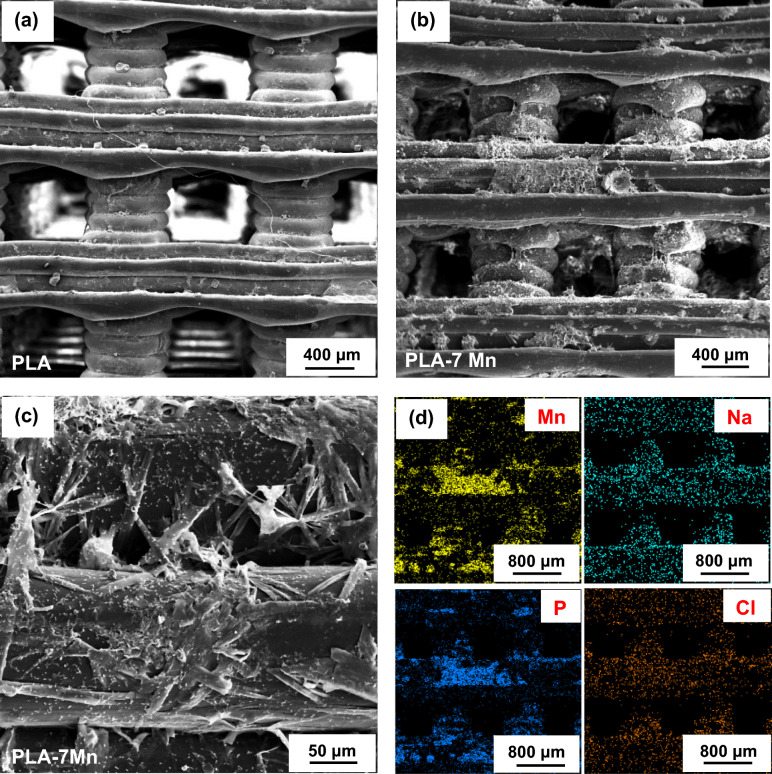
FESEM micrographs of PLA (**a**) and PLA-7Mn (**b**, **c**) scaffolds after 4 weeks of immersion in the PBS solution at 37 °C. The corresponding EDS mapping analysis of (**b**) is presented in (**d**) for the elements of Mn, Na, P, and Cl.

To understand better the degradation and bio-mineralization behavior of the studied samples, the surface morphologies of the PLA and PLA-7Mn scaffolds after 4 weeks of immersion were studied by FESEM and the corresponding results are depicted in Fig. 14. It is clear that a higher amount of corrosion products accumulated on the struts and between the layers of the composite sample in comparison with pure PLA (Fig. 14a–c). The positive ions in the PBS solution, such as Na^+^, can be attracted to the hydroxyl and carboxyl groups of PLA. Thus, the Cl^-^ in solution can react with this positive ion to form NaCl, which will be deposited on the scaffold surface. Also, the negative ion of (PO4)^3−^ can be attracted to the deposited positive charges of filler, and phosphate-based compounds can be crystalized on the sample surface^56^. Owing to the higher number of the mentioned functional groups in the composite PLA-Mn scaffolds, as discussed before in the DSC analysis, and also the rougher surface of these scaffolds as a suitable platform for apatite nucleation, a higher amount of apatite containing NaCl, would be precipitated on the surface, according to the EDS analysis shown in Fig. 14d. Furthermore, a high concentration of Mn and P appeared on the precipitated apatite (Fig. 14d). In this regard, it has been reported that^57,58^ the (PO4)^3−^ group can promote Mn release during the degradation process and produce a higher amount of precipitation. It can also confirm that the biodegradation products may consist of Mn^+2^ and Mn-phosphate and oxide-based compounds. Nevertheless, further study is needed to determine the exact composition of the corrosion products. In conclusion, it can be supposed that the PLA-7Mn scaffolds would have better bioactivity due to higher crystallization of bone-composition apatite on the surface of the composite scaffolds^59^.

**Figure 15:**
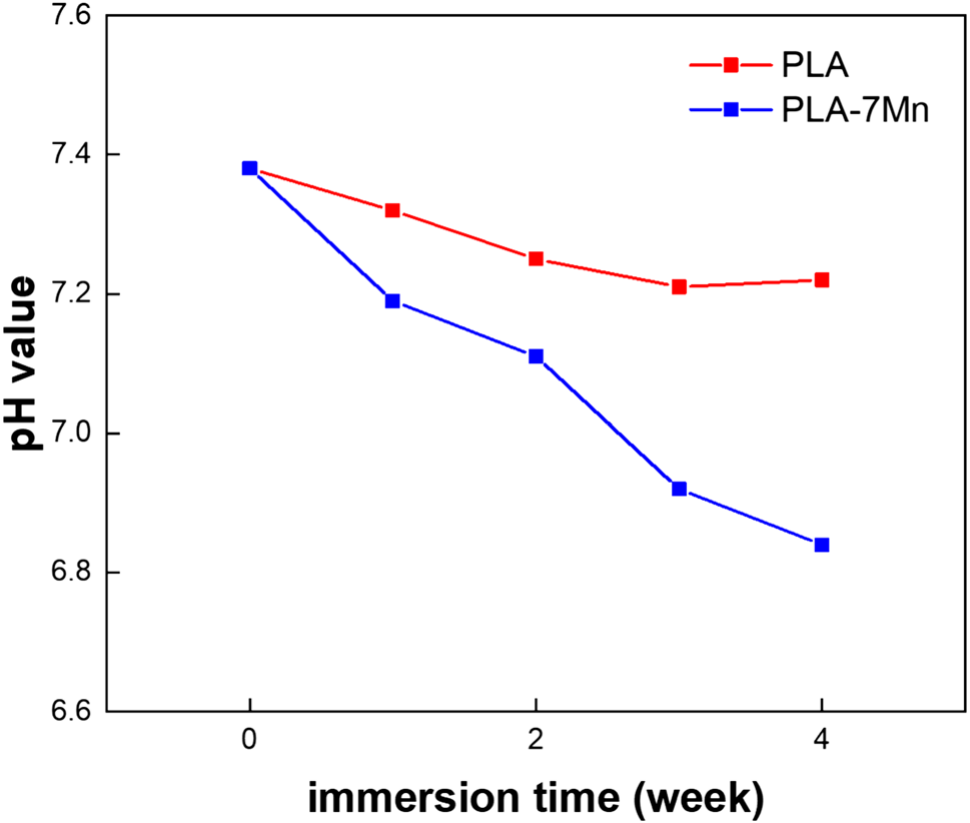
Variations of pH with immersion time for pure PLA and PLA-7Mn scaffolds.

Variations of measured pH values of the solution with immersion time are shown in Fig. 15 for the pure PLA and PLA-7Mn scaffolds. A similar downward trend can be observed both scaffolds, however, the slope of variations is more severe in the case of the PLA-7Mn scaffold, as pH decreased from 7.38 to 6.84 after 4 weeks of immersion.

Generally, the hydrolysis reaction of PLA and breakage of Ester bonds make the immersion medium acidic:

4 $${\text{Biopolymer }} + {\text{ H}}_{{2}} {\text{O }} \to {\text{R}}_{{1}} - {\text{ COOH }} + {\text{ R}}_{{2}} - {\text{ OH}}$$

Decrease in pH value in PBS typically occurs when the molecular weight of PLA reaches a critical value, allowing for the formation of soluble polymer chains. This phenomenon can lead to the acceleration of the PLA hydrolysis process. The formation of phosphate and hydroxide of manganese are likely to decrease the pH of PBS, mainly due to the reduction in phosphate ions affecting the buffer equilibrium and potential hydrolysis of Mn^2+^ ions releasing H^+^ ions^57^. Also, addition of Mn particles accelerated the degradation rate of the composite as discussed before in this research, thereby increasing the hydrolysis rate of PLA and subsequently reducing the pH of the medium.

**Figure 16:**
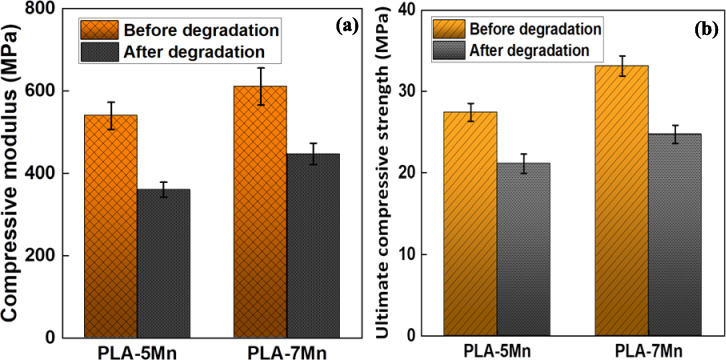
Compression test results on scaffolds after 10 weeks of degradation: (**a**) compressive modulus, and (**b**) ultimate compressive strength of PLA-5Mn and PLA-7Mn scaffolds.

To understand the mechanical response of degraded scaffolds, compression test was conducted on PLA-7Mn and PLA-5Mn scaffolds (chosen as optimum samples in terms of mechanical properties) after degradation tests in the PBS solution for 10 weeks, and the obtained results are illustrated in Figs. S2 and 16. Following 10 weeks of degradation, the compressive modulus of PLA-7Mn and PLA-5Mn scaffolds declined significantly from 610 and 539 MPa to 447 and 361 MPa, corresponding to 27 and 33% reduction, respectively (Fig. 16a). The same trend is pronounced in the case of UCS of both samples and they witnessed approximately 30% decrease (Fig. 16b).

#### Cell viability

**Figure 17:**
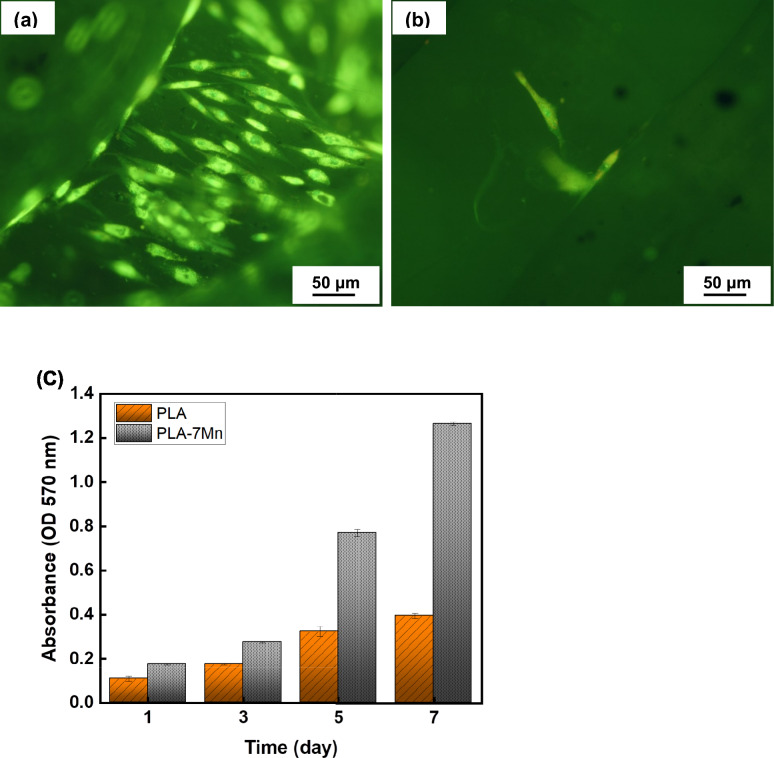
Cell viability results for the neat PLA and PLA-7Mn scaffolds. Fluorescent imaging of live (green) and dead (red) cells on the surface of PLA-7Mn (**a**) and neat PLA (**b**) scaffolds. The ratio of live/dead cells at intervals of 1, 3, 5 and 7 days are shown in (**c**).

Cell viability and proliferation of neat PLA and PLA-7Mn scaffolds were evaluated via the MTT assay technique and the obtained results are presented in Fig. 17. According to fluorescence images, a noticeable number of live cells (green color) covers the PLA-7Mn scaffold (Fig. 17a), while just few live cells exist on the PLA scaffold (Fig. 17b). Additionally, quantitative characterization of cell proliferation and cytotoxicity is demonstrated in Fig. 17c, where it is clear that by increasing the incubation time, cell proliferation level of the PLA-7Mn scaffold increases significantly compared to the neat PLA scaffold. Accordingly, it can be concluded that incorporation of Mn enhances significantly the cytocompatibility of PLA scaffolds.

A number of factors can be proposed for the observed improved cytocompatibility. In general, it has been reported that Mn is naturally biocompatible and promotes cells multiplication^16,60^. In addition, surface roughness and hydrophilicity of scaffolds significantly influence cell attachment properties^61^. In this regard and as the data presented in previous parts confirm, the surface roughness and hydrophilicity of PLA-7Mn scaffold surpasses that of the PLA scaffold, thus incorporation of Mn particles to PLA increase the cell viability of samples.

### A comparison between PLA-7Mn and other PLA-Metal composites

A succinct comparative analysis between mechanical properties, wettability and cell viability of PLA-7Mn and some other PLA-Metal composites is presented in Table 3, where each category highlights the optimal metal loading. Notably, PLA-1Gr-1Mg^52^ and PLA-10 vol% 316L^15,47^ exhibit superior compressive strength and modulus compared with PLA-7Mn, while mechanical properties of PLA-6Mg^10^ and PLA-5Ti-5PEG^19^ is weaker than the PLA-7Mn. Remarkably, PLA-7Mn, boasting a contact angle of 73.4°, emerges as the second-most favorable scaffold in terms of wettability.

**Table 3:** Brief comparison between properties of PLA-7Mn and some other PLA-metal composites.

Composite	Mechanical properties		Contact angle (°)	Cell viability after 5 days (Absorbance OD nm)
	Compressive strength (MPa)	Compressive modulus (GPa)		
PLA-7 wt% Mn	33.1	0.611	73.4	0.77 (570 nm)
Neat PLA	15.7	0.457	94.1	0.32 (570 nm)
PLA-5 wt% Ti-5 wt% PEG^19^	29.3	0.490	65.6	0.14 (570 nm)
PLA-1 wt%Gr-1 wt% Mg^52^	50.0	–	87.6	–
PLA-10 vol% (~ 41.3 wt%) Fe^47^	30.0	1.000	70.9	–
PLA-10 vol % (~ 41.7 wt%) 316L^15,47^	39.1	1.510	96.5	–
PLA-6 wt% Mg^10^	19.7	0.575	88.0	0.16 (570 nm)

## Conclusions

In this investigation, PLA-*x*Mn composite scaffolds (*x* = 0, 1, 3, 5 and 7 wt%) were 3D printed via the FDM method. The most important conclusions can be summarized as follows.

1. The presence of Mn particles increased the chain mobility and flowability of PLA, which was attributed to the metal-catalyzed depolymerization. This phenomenon reduced the dimensional accuracy of printed PLA-7Mn scaffolds, but improved the interlayer adhesion.
2. According to the DSC results, *T*_cc_ was declined by 6 ℃, whereas the degree of total crystallinity was increased as the result of Mn addition. Moreover, the *T*_g_ and *T*_m_ were unaffected.
3. The FESEM micrographs illustrated uniform distribution of Mn particles through the composite filaments. In addition, surface roughness of the filaments was increased as the result of Mn addition.
4. Mn addition noticeably enhanced the mechanical properties. The PLA-7Mn scaffold demonstrated the highest compressive modulus and ultimate compressive strength of 611 MPa and 33.1 MPa respectively, which was mainly attributed to the high reinforcing ability of Mn and also better interlayer adhesion in the composite scaffolds.
5. The wettability and water absorption capacity of PLA scaffold increased with Mn addition, owing to the increased surface roughness and polarity of the scaffold surface.
6. Mn addition significantly enhanced the degradation rate and bio-mineralization ability of PLA scaffolds.
7. The ratio of live/dead cells in PLA-Mn scaffold experienced a threefold increase in comparison with neat PLA which demonstrates improved biocompatibility with the incorporation of Mn particles.

### Statistical analysis

All graphs and statistical analyses were prepared using Origin software (version 2018). Each test was repeated at least three times, and results were reported as mean ± standard deviation (SD).

### Supplementary Information

Supplementary Figures.

## Data Availability

The datasets generated during and/or analyzed during the current study will be available upon reasonable request from the corresponding author.
