# Supplementary material for: Fabrication, characterization and evaluating properties of 3D printed PLA-Mn scaffolds
Source: Sci Rep. 2024 Jul 18;14:16592. doi: 10.1038/s41598-024-67478-9 (PMC11258323; doi:10.1038/s41598-024-67478-9)
Supplement: Supplementary file 1 — Supplementary Figures. [file 41598_2024_67478_MOESM1_ESM.docx]

Supporting Information

**Fabrication, characterization and evaluating properties of 3D printed PLA-Mn scaffolds**

Sina Dehghan-Toranposhti^a,b^, Rasoul Bakhshi^a^, Reza Alizadeh^a,*^, Mahboubeh Bohlouli^c,**^

^a^*Department of Materials Science and Engineering, Sharif University of Technology, Tehran, Iran*

^b^ *Department of Materials Science and Engineering, North Carolina State University, Raleigh, NC, USA*

^c^*Department of Tissue Engineering and Applied Cell Sciences, School of Advanced Technologies in Medicine, Shahid Beheshti University of Medical Sciences, Tehran, Iran*

*Main corresponding author’s contact details:

Reza Alizadeh, Ph.D.

Assistant professor

Sharif University of Technology, Azadi Ave., Tehran 11155-9466, Iran.

Email: [r.alizadeh@sharif.edu](mailto:r.alizadeh@sharif.edu)

**Co-corresponding author’s contact details:

Mahboubeh Bohlouli, Ph.D.

Assistant professor

Email: [bohlouli98@gmail.com](mailto:bohlouli98@gmail.com)


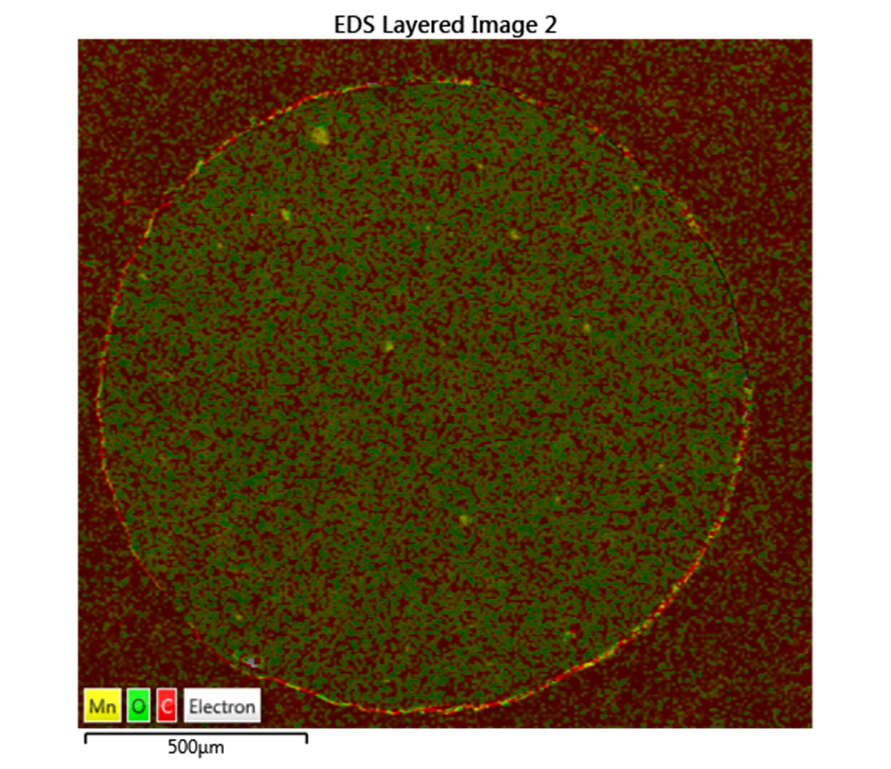

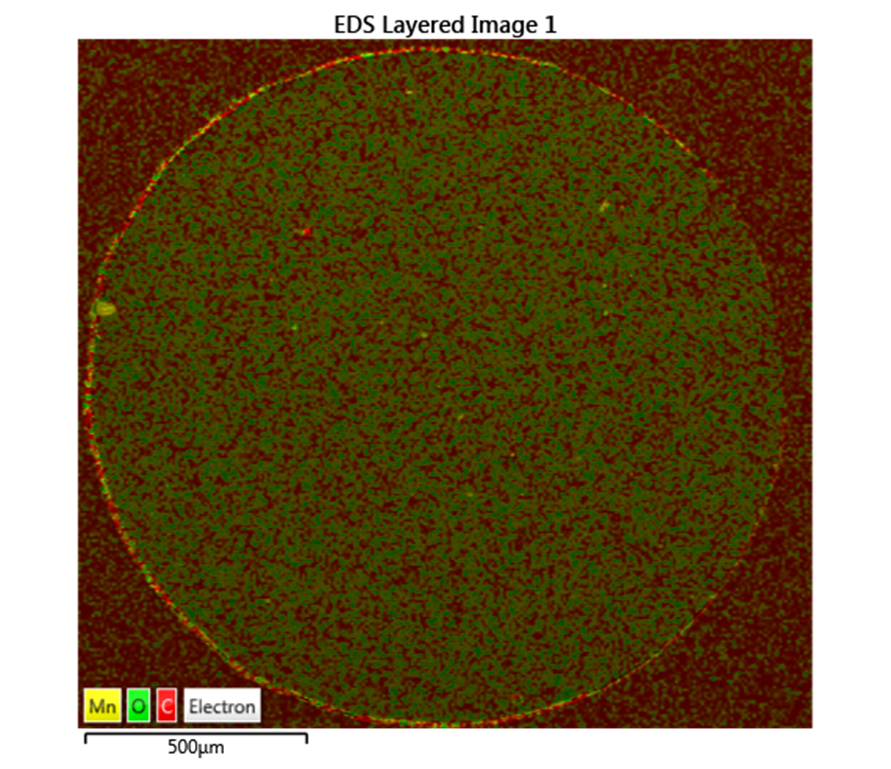

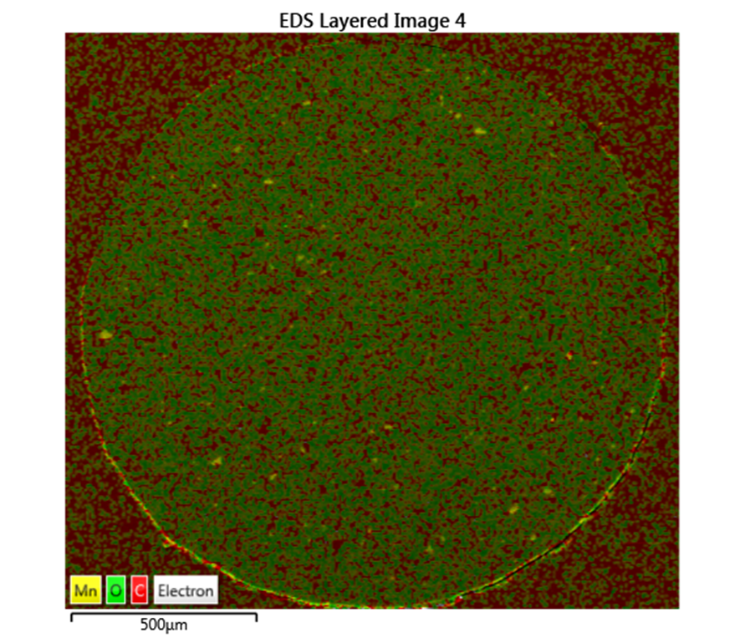

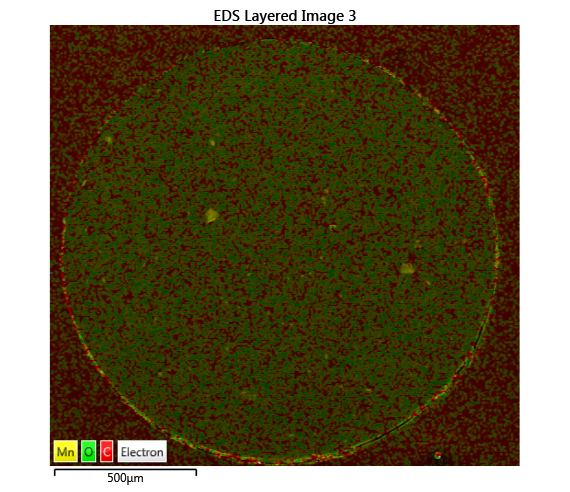


**(a)**

**(b)**

**(c) (a)**

**(d) (a)**

**PLA-3Mn**

**PLA-1Mn**

**PLA-5Mn**

**PLA-7Mn**

**Fig. S1**. EDS map analysis from cross section of: (a) PLA-1Mn, (b) PLA-3Mn, (c) PLA-5Mn, (d) PLA-7Mn composite filaments.


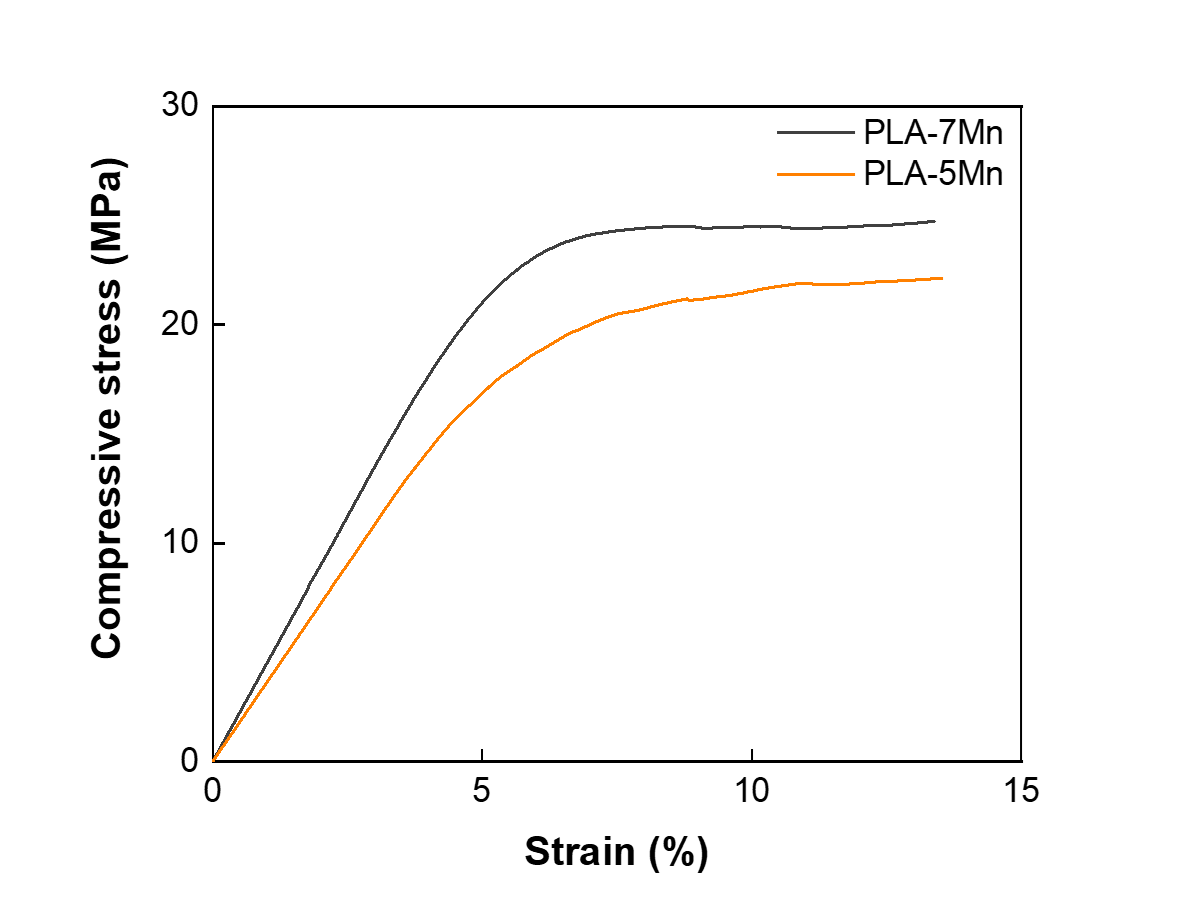


**Fig. S2**. Compression stress-strain curves of the PLA-5 and PLA-7Mn scaffolds after degradation tests for up to 10 weeks.
